# Rates, costs and determinants of lumbar spine imaging in population-based women born in 1973–1978: Data from the Australian Longitudinal Study on Women’s Health

**DOI:** 10.1371/journal.pone.0243282

**Published:** 2020-12-03

**Authors:** Yuanyuan Wang, Sultana Monira Hussain, Anita E. Wluka, Yuan Z. Lim, Donna M. Urquhart, Gita D. Mishra, Helena Teede, Jenny Doust, Wendy J. Brown, Flavia M. Cicuttini

**Affiliations:** 1 Department of Epidemiology and Preventive Medicine, School of Public Health and Preventive Medicine, Monash University, Melbourne, Victoria, Australia; 2 School of Public Health, University of Queensland, Brisbane, Queensland, Australia; 3 Monash Centre for Health Research and Implementation, School of Public Health and Preventive Medicine, Monash University, Victoria, Australia; 4 Diabetes and Vascular Medicine Unit, Monash Health, Clayton, Victoria, Australia; 5 Faculty of Health Sciences and Medicine, Bond University, Gold Coast, Queensland, Australia; 6 School of Human Movement and Nutrition Sciences, University of Queensland, Brisbane, Queensland, Australia; Kaiser Permanente Washington, UNITED STATES

## Abstract

**Objective:**

There are concerns that lumbar spine imaging represents low value care. Our aim was to examine the use of lumbar spine imaging [radiography, computed tomography (CT), magnetic resonance imaging (MRI)] over 20 years, and costs and person-level characteristics of imaging in a large cohort of Australian women.

**Methods:**

The Australian Longitudinal Study on Women’s Health (ALSWH) is a longitudinal population-based survey of women randomly selected from national health insurance scheme (Medicare) database. This study examined 13458 women born in 1973–1978 who consented to link their ALSWH and Medical Benefits Scheme records. Self-reported data on demographics, body mass index, depression, physical and mental health, and back pain were collected in each survey performed in 1996, 2000, 2003, 2006, 2009, 2012, and 2015. Data on lumbar spine imaging from 1996 to 2015 were obtained from the Medical Benefits Scheme database.

**Results:**

38.9% of women underwent some form of lumbar spine imaging over 20 years. While radiography increased from 1996 to 2011 and decreased thereafter, CT and MRI continued to increase from 1996 to 2015. In women with self-reported back pain, depression and poorer physical health were associated with imaging, with no significant differences in types of imaging. Based on imaging rates in ALSWH, the estimated costs for Australian women aged 30–39 years were AU$51,735,649 over 2011–2015.

**Conclusions:**

Lumbar spine imaging was common in population-based Australian women, with rates increasing over 20 years. Depression and poor physical health were associated with lumbar spine imaging. Raising awareness of this in clinicians is likely to result in significant cost savings if clinical guidelines are followed, with the potential of freeing resources for high value care and health outcomes.

## Introduction

Low back pain (LBP), the leading cause of disability worldwide, is a common reason for seeking medical care and associated with substantial direct and indirect costs [1]. Non-specific LBP, defined as LBP not attributable to a known cause, represents 90–95% of the LBP cases [2]. Of concern is the overuse of lumbar spine imaging, including radiography, computed tomography (CT) and magnetic resonance imaging (MRI). A meta-analysis of over 4 million imaging requests across 21 years found one in four patients presenting to primary care with LBP received imaging, and complex imaging increased by over 50% from 1995 to 2015 [3]. This is despite evidence-based clinical guidelines [4–7] and Choosing Wisely campaigns [8–10] recommending against the use of routine diagnostic imaging for patients with non-specific LBP in the absence of clinical red flags (i.e. severe or progressive neurologic deficits or features suggesting a serious or specific underlying condition such as fracture, malignancy, cauda equine syndrome, or infection), thus representing low value care. Imaging for LBP has been listed in the evidence-based “Top 5” activities in primary care practice where change in practice could improve the quality of care and use of clinical resources [11]. Routine diagnostic lumbar spine imaging, in the absence of indications of serious underlying conditions, provides no benefit to clinical outcomes [12, 13], while exposing patients to radiation, unnecessary additional tests and ineffective treatments, and increasing healthcare costs [14, 15]. Various interventions have been tested to reduce the rate of lumbar spine imaging, but with limited effect [16].

There is little information about imaging utilization and its associated costs in community-based populations, with most work having been performed in clinical populations with LBP [17, 18]. Understanding this will be important for improving clinical decision making, optimising health resources, and providing valuable information about low value care in the general population. As women have more back pain and utilise more healthcare resources than men [19–21], we examined the use of lumbar spine imaging in population-based Australian women born in 1973–1978 over 20 years, costs and person-level characteristics associated with imaging, and estimated imaging costs for Australian women of the same age group. The Australian Longitudinal Study on Women’s Health (ALSWH) was chosen because it is a largely representative sample of the general population of Australian women [22–24].

## Materials and methods

### Participants

The ALSWH (www.alswh.org.au) is a longitudinal population-based survey of over 44,000 women randomly selected from the national health insurance scheme (Medicare) database which includes most permanent residents of Australia, with intentional oversampling from rural and remote areas [22, 25]. The study first collected mailed survey data from three age cohorts in 1996: 1973–1978 cohort, 1946–1951 cohort, and 1921–1926 cohort. The surveys included questions about a diverse range of issues including health behaviours, health service use, physical and mental health, social and demographic factors. The Human Research Ethics Committees of the University of Newcastle (H-076-0795, H-2011-0371) and Australian Department of Health (11/2008) approved the study protocol. Written informed consent was obtained from all participants. The 1973–1978 cohort (n = 14247) completed surveys in 1996, 2000, 2003, 2006, 2009, 2012, and 2015. Consent was sought from all participants for Medicare Australia to release linkable claim details to the research team. The current study included women who consented to the linkage of their ALSWH and Medical Benefits Scheme (MBS) records (n = 13458).

### Lumbar spine imaging

All Australian citizens and permanent residents, including those without private health insurance, have access to quality health care service under Medicare. MBS items for lumbar spine imaging (radiography, CT, and MRI) were identified from the MBS Book from 1996 to 2015 which provides information on payment of Medicare benefits for professional services rendered by registered medical practitioners [26].

### Demographics

Education levels were categorised as no formal qualifications to higher school certificate, and trade/apprenticeship/certificate/diploma/university degree or higher [27]. Working status was classified as full-time paid work, part-time or casual paid work, and no paid work [27]. Depression was assessed by the question “Have you ever been told by a doctor that you have depression (not postnatal)?” [27].

### Body mass index

Body mass index (BMI) was calculated from self-reported height and weight [27] and obesity defined by BMI ≥30.0 kg/m^2^.

### Physical and mental health

Physical and mental health were assessed using the 36-item Short Form Health Survey (SF-36) [28]. Physical component summary (PCS) and mental component summary (MCS) scores were calculated (0–100), with higher scores indicating better health.

### Back pain

For the question “In the last 12 months have you had back pain?” women were asked to circle one response for back pain frequency: ‘never’, ‘rarely’, ‘sometimes’ or ‘often’. Those who responded ‘never’ or ‘rarely’ were categorized as ‘no back pain’, those who responded ‘sometimes’ or ‘often’ were categorized as ‘back pain’ [27].

### Statistical analysis

Descriptive characteristics of imaging use were presented, for the whole study period (1996–2015), every 5 years (Period 1: 1996–2000; Period 2: 2001–2005; Period 3: 2006–2010; Period 4: 2011–2015), and each calendar year. To explore whether the change in imaging use from 1996–2000 to 2011–2015 was due to ageing of the cohort or the effect of time period, we categorised the women into 5-year age groups, i.e. from 16–20 and 21–25 years old in 1996 to 31–35 and 36–40 years old in 2011, and examined imaging use over each 5-year time period. Costs of imaging from 1996–2015 were calculated based on the type and number of MBS items received and cost of each MBS item in the most recent MBS book. We calculated costs of imaging for the most recent two time periods (2006–2015) for Australian women population of the same age group, based on Australian women population aged 25–34 years in 2006 [29], as the patterns of imaging use over these two time periods better reflected the change in availability of imaging facilities for lumbar spine, particularly MRI. Participant characteristics were compared between women with self-reported back pain with and without lumbar spine imaging using independent samples t tests and chi-square tests, and binary logistic regression was used for adjustment for these characteristics. All analyses were performed using Stata SE version 14.0 (StataCorp).

## Results

**Fig 1:**
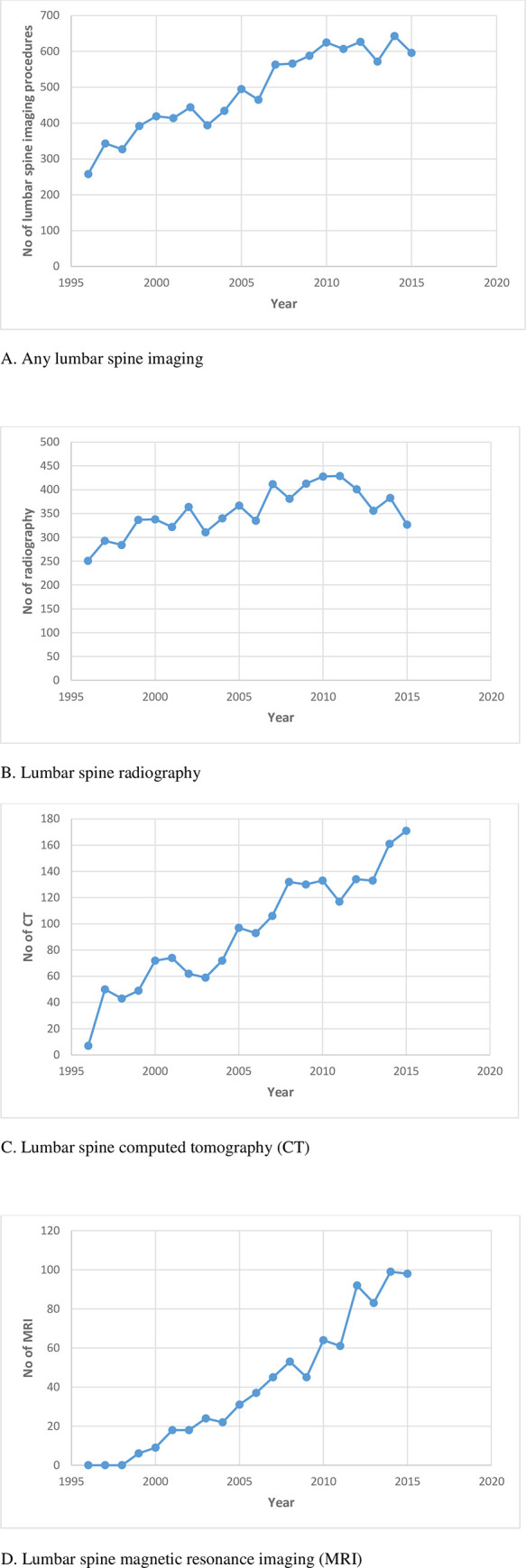
Number of lumbar spine imaging procedures per year from 1996 to 2015.

The number of all imaging procedures per year increased from 1996 to 2010 (2.4 times), stabilizing between 2010 and 2015 (Fig 1A). Radiography increased by 1.7-fold from 1996 to 2011, reducing thereafter (Fig 1B). CT increased from 1996 to 2015 (24.4 times) (Fig 1C), and MRI increased from 1999 (no MRI prior to 1999) to 2015 (16.3 times) (Fig 1D). Table 1 presents the number of imaging procedures (original data for Fig 1) and number of women receiving imaging per year from 1996 to 2015. The trend of change in the annual percentage of women who underwent at least one imaging procedure (any imaging and by type) was similar to the trend of change in imaging procedures over the study period. There is also a trend for increasing numbers of women who received multiple imaging.

**Table 1:** Annual lumbar spine imaging from 1996 to 2015 among 13458 women.

	1996	1997	1998	1999	2000	2001	2002	2003	2004	2005	2006	2007	2008	2009	2010	2011	2012	2013	2014	2015
**No of imaging procedures**																				
Any imaging	258	343	327	392	419	414	444	394	434	495	465	563	566	588	625	607	627	572	643	596
Radiography	251	293	284	337	338	322	364	311	340	367	335	412	381	413	428	429	401	356	383	327
CT	7	50	43	49	72	74	62	59	72	97	93	106	132	130	133	117	134	133	161	171
MRI	0	0	0	6	9	18	18	24	22	31	37	45	53	45	64	61	92	83	99	98
**No of women being imaged**																				
Any imaging	250	303	298	349	378	363	387	350	370	421	405	475	483	512	542	530	555	491	556	517
Radiography	246	275	273	321	327	310	345	301	321	355	318	389	367	402	415	415	385	343	368	311
CT	4	47	43	46	70	72	61	57	69	90	88	103	122	125	130	116	130	125	157	166
MRI	0	0	0	6	9	18	18	21	20	25	34	39	48	41	55	57	84	72	92	92
**Multiple imaging procedures, no of women**																				
>2 imaging procedures	5	26	27	38	40	42	48	40	48	59	46	65	64	58	62	62	61	61	68	63
>3 imaging procedures	2	4	2	5	1	6	6	4	10	11	7	15	14	11	10	12	10	14	15	13
**Patterns of imaging procedures, no of women**																				
Radiography only	246	256	255	300	299	278	311	276	285	315	292	345	324	357	369	365	349	308	318	272
Radiography & CT	0	19	18	20	26	25	30	19	33	31	22	35	29	33	32	41	22	21	36	23
Radiography & MRI	0	0	0	0	2	4	2	5	1	4	2	3	8	6	10	5	12	8	11	13
Radiography & CT & MRI	0	0	0	1	0	3	2	1	2	5	2	6	6	6	4	4	2	6	3	3
CT only	4	28	25	23	44	42	28	34	32	50	57	56	82	81	86	67	100	90	110	130
CT & MRI	0	0	0	2	0	2	1	3	2	4	7	6	5	5	8	4	6	8	8	10
MRI only	0	0	0	3	7	9	13	12	15	12	23	24	29	24	33	44	64	50	70	66

CT: computed tomography; MRI: magnetic resonance imaging.

During 1996–2015, 5237 (38.9%) women had some form of lumbar spine imaging (Table 2). While the numbers of women having CT and MRI increased from 1996 to 2015, the numbers of women having radiography increased from 1996 to 2010 then reduced in 2011–2015. Radiography only was most common (n = 3524, 67.3%), followed by radiography and CT (n = 752, 14.4%), CT only (n = 405, 7.7%), radiography, CT and MRI (n = 205, 3.9%), radiography and MRI (n = 167, 3.2%), MRI only (n = 133, 2.5%), CT and MRI (n = 51, 1.0%). The number of women receiving multiple types of imaging increased over the study period. S1 Table presents the numbers of women receiving different numbers of imaging over the study period.

**Table 2:** Use of lumbar spine imaging from 1996 to 2015.

	Whole study period	Specific time periods			
	1996–2015, no (%)	Period 1	Period 2	Period 3	Period 4
		1996–2000, no (%)	2001–2005, no (%)	2006–2010, no (%)	2011–2015, no (%)
Women being imaged	5237 (38.9)	1436 (10.7)	1644 (12.2)	2058 (15.3)	2152 (16.0)
Radiography	4648 (34.5)	1332 (9.9)	1470 (10.9)	1692 (12.6)	1618 (12.0)
CT	1413 (10.5)	207 (1.5)	326 (2.4)	517 (3.8)	616 (4.6)
MRI	556 (4.1)	15 (0.1)	93 (0.7)	190 (1.4)	326 (2.4)
**Patterns of imaging use**					
Radiography only	3524 (67.3)	1220 (85.0)	1260 (76.6)	1426 (69.3)	1313 (61.0)
CT only	405 (7.7)	94 (6.5)	128 (7.8)	259 (12.6)	320 (14.9)
MRI only	133 (2.5)	7 (0.5)	34 (2.1)	76 (3.7)	160 (7.4)
Radiography and CT	752 (14.4)	107 (7.5)	163 (9.9)	183 (8.9)	193 (9.0)
Radiography and MRI	167 (3.2)	2 (0.1)	24 (1.5)	39 (1.9)	63 (2.9)
CT and MRI	51 (1.0)	3 (0.2)	12 (0.7)	31 (1.5)	54 (2.5)
Radiography, CT, and MRI	205 (3.9)	3 (0.2)	23 (1.4)	44 (2.1)	49 (2.3)

CT: computed tomography; MRI: magnetic resonance imaging.

**Fig 2:**
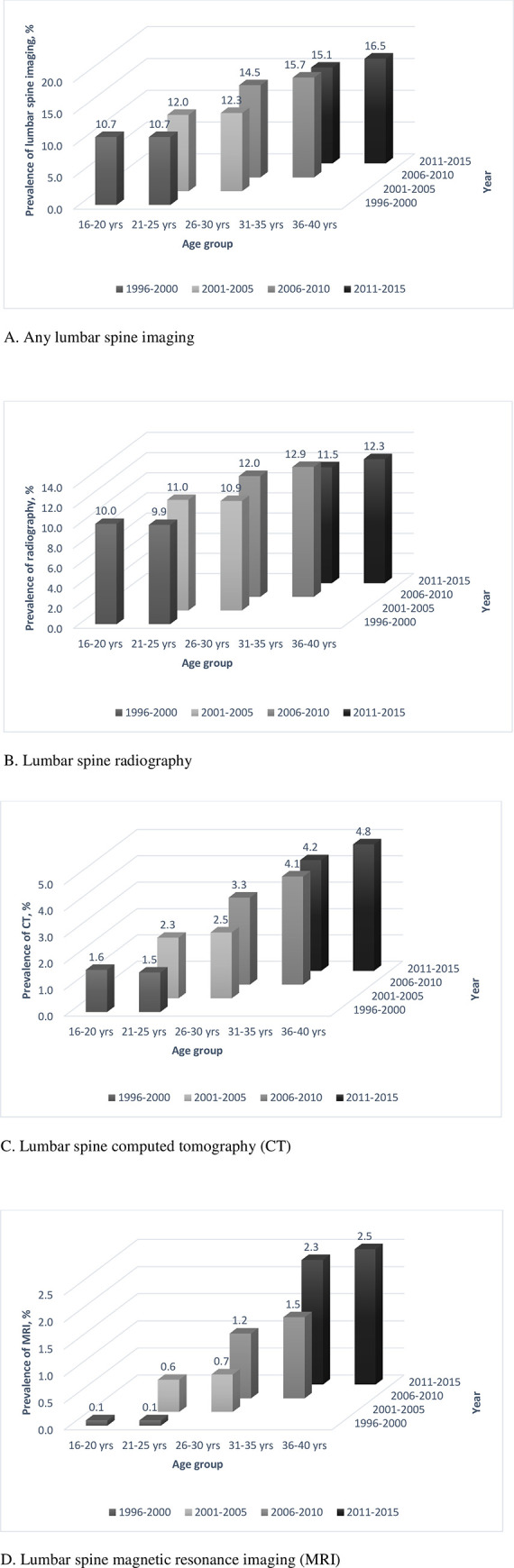
Prevalence of lumbar spine imaging by age group and time period.

Fig 2 shows imaging use over each 5-year time period in women who were categorised into 5-year age groups. Rates of imaging increased from 1996–2000, 2001–2005, to 2006–2010, then stabilised in 2011–2015 (Fig 2A). Rates of radiography and CT increased from 1996–2000, 2001–2005, to 2006–2010, then levelled off for radiography and stabilised for CT in 2011–2015 (Fig 2B and 2C). Rates of MRI increased over all time periods (Fig 2D). These data indicated a time period effect of increasing imaging. At each time period from 2006–2010, older women had higher rates of imaging (Fig 2A–2D), showing an ageing effect on increasing imaging.

Costs of lumbar spine imaging for the study population are shown in Table 3. Costs for radiography, CT, and MRI during 2006–2015 were AU$363,514, AU$314,906, and AU$257,331, respectively (total AU$935,751). If Australian women aged 25–34 years underwent imaging at the rates in ALSWH, based on the Australian female population aged 25–34 years in 2006 (n = 1,355,334), the estimated costs in 2006–2015 would be AU$36,607,654 for radiography, AU$31,712,588 for CT, and AU$25,914,501 for MRI (total AU$94,234,843).

**Table 3:** Costs of lumbar spine imaging in the ALWSH cohort from 1996 to 2015 and cost estimates from 2006 to 2015 for Australian women aged 25–34 years in 2006, AU$.

	ALSWH					Australian women		
	1996–2000	2001–2005	2006–2010	2011–2015	2006–2015	2006–2010	2011–2015	2006–2015
Radiography	136680	160684	187979	175535	363514	18930413	17677241	36607654
CT	53597	87323	142646	172260	314906	14365156	17347432	31712588
MRI	7125	49665	91392	165939	257331	9203625	16710876	25914501
Total	197402	297672	422017	513734	935751	42499194	51735649	94234843

ALSWH: the Australian Longitudinal Study on Women’s Health; CT: computed tomography; MRI: magnetic resonance imaging.

There were some changes over the 20-year period in BMI (22.7 kg/m^2^ in 1996 and 27.0 kg/m^2^ in 2015), education (29.0% in 1996 and 84.8% in 2015 for trade/apprenticeship/certificate/diploma/university degree or higher), and MCS (43.5 in 1996 and 45.6 in 2015). There were no significant differences in baseline characteristics (age, BMI, PCS, MCS, depression, education, or working status) between the women who attended survey 6 (2012, n = 7510) and those who did not. In those who reported back pain in the last 12 months in survey 6 (n = 3739), we compared the characteristics of women with and without lumbar spine imaging performed in the preceding year (Table 4). Women receiving imaging were more likely to have depression and had lower PCS than those without imaging, with no significant differences between those receiving different imaging modalities. Depression and lower PCS score were associated with imaging in multivariable analysis.

**Table 4:** Participant characteristics and use of lumbar spine imaging in participants with self-reported back pain in the last 12 months.

	Any imaging* N = 223	Radiography N = 168	CT N = 54	MRI N = 26	No imaging* N = 3516	P*	Multivariable analysis^⁋^	
							Odds ratio (95% CI)	P
Age, years	36.8 (1.5)	36.8 (1.5)	37.1 (1.5)	36.6 (1.4)	36.8 (1.5)	0.62	1.04 (0.94, 1.14)	0.47
Body mass index, kg/m^2^	27.3 (7.0)	27.0 (7.0)	28.5 (7.0)	26.7 (5.1)	27.0 (6.6)	0.46	0.99 (0.97, 1.01)	0.28
Obesity, n (%)	59 (27.1)	42 (25.6)	15 (27.8)	6 (24.0)	876 (25.4)	0.58	0.86 (0.62, 1.18)	0.35
Depression, n (%)	64 (29.1)	44 (26.5)	18 (33.3)	13 (52.0)	680 (19.7)	0.001	1.55 (1.09, 2.20)	0.01
SF-36 PCS	46.3 (11.6)	47.2 (11.4)	45.5 (12.1)	42.1 (10.4)	50.1 (8.7)	<0.0001	0.96 (0.95, 0.98)	<0.001
SF-36 MCS	44.3 (11.7)	44.4 (11.9)	44.1 (12.7)	41.4 (11.9)	45.1 (11.3)	0.33	1.00 (0.98, 1.01)	0.50
Trade/apprenticeship/ certificate/diploma/university degree or higher, n (%)	175 (79.2)	138 (83.1)	36 (66.7)	22 (84.6)	2803 (81.0)	0.51	0.92 (0.65, 1.31)	0.65
Working status, n (%)						0.74		
Full-time paid work	78 (35.3)	64 (38.6)	16 (29.6)	7 (26.9)	1170 (33.7)		1.00	
Part-time or casual paid work	82 (37.1)	57 (34.3)	21 (38.9)	12 (46.2)	1381 (39.7)		0.88 (0.63, 1.22)	0.45
No paid work	61 (27.6)	45 (27.1)	17 (31.5)	7 (26.9)	926 (26.6)		0.87 (0.61, 1.26)	0.47

CT: computed tomography; MRI: magnetic resonance imaging; PCS: physical component summary; MCS: mental component summary; CI: confidence interval.

Data on participant characteristics were from the 6th survey (2012) and presented as mean (standard deviation) or n (%).

*For difference between women with and without lumbar spine imaging.

^⁋^Any imaging vs. no imaging, including all variables in the same logistic regression model.

## Discussion

In population-based Australian women born in 1973–1978, 38.9% underwent some form of lumbar spine imaging over 20 years. Although radiography remained the most common procedure, its use decreased after 2011, and the use of CT and MRI increased over the 20 years. The increased imaging rates were not simply due to ageing, but also time period related factors. Costs of lumbar spine imaging were estimated over 51 million Australian dollars for Australian female population aged 30–39 years over 5 years. Depression and poor physical health were associated with imaging in women with back pain, with no significant difference in types of imaging.

In primary care settings about 25% of patients with a new episode of LBP undergo imaging [3, 30, 31]. In our study 38.9% women underwent some form of imaging from 1996 to 2015, with annual rate increasing from 1.9% [250/13458] in 1996 to 4.1% [556/13458] in 2014 (Table 1). Overall lumbar spine imaging increased from 1996 to 2010, stabilising between 2010 and 2015. This was due to a slight reduction in radiography since 2011, but a continued increase in CT and MRI and more women receiving multiple imaging. The overall increase in imaging in our community-based study was consistent with findings from studies of patients with LBP [17, 18, 30], despite clinical guidelines discouraging routine imaging. Data from our study and others [3, 17, 18] demonstrated a significant rapid increase in expensive, advanced imaging, discordant with current guidelines [4–7].

Clinical guidelines recommend imaging where red flags are present [4–7]. In primary care settings, the rates of serious underlying pathology identified by diagnostic imaging are very low: fracture (4%), malignancy (0.7%), infection (0.01%), axial spondyloarthritis (0.1–1.4%), and cauda equine syndrome (0.04%) [32, 33]. In those aged under 50 years, fracture and malignancy are even less common. In an Australian study of 1172 consecutive patients presenting to primary care clinics with acute LBP, although 80% had at least one red flag, only 0.9% had a serious underlying pathology [34]. This study showed that combination of positive red flag features yielded a sensitivity of 38% and a specificity of 100% to identify serious pathology [34], with similar findings observed in studies from other countries [35, 36]. Thus based on these data, if clinical guidelines for lumbar spine imaging are followed, it is likely that approximately three LBP patients with red flags would be imaged to detect one case of serious pathology in primary care settings. In the ALSWH cohort, the proportions of women who reported “often” back pain and seeking help for back pain (i.e. those presenting to primary care and other health professionals) was 9.1% at survey 6 (2012) and 9.3% at survey 7 (2015). Therefore, the average, 9.2% per annum, was taken as the estimate of the prevalence during 2011–2015. Thus 34 (13458*9.2%*0.9%*3) women in our study would have received imaging each year as recommended by clinical guidelines [4–7]. Our estimate is likely to be overestimated as the ALSWH is a community-based population of younger age. If these women had one MRI each year, the estimated imaging costs over 5 years (2011–2015) would be AU$68,000 ($400*34*5), corresponding to AU$6,848,173 for Australian women of the same age over this 5 year period. Thus, there would be a potential cost saving of over 44 million Australian dollars in lumbar spine imaging over 5 years (2011–2015) in this population alone if clinical guidelines are followed.

Clinical guidelines recommend radiography only for initial evaluation of LBP in patients with a history of low-velocity trauma, osteoporosis, or chronic steroid use [14, 37]. If a red flag is present, MRI is preferred over CT, but CT can be performed if MRI is contraindicated or unavailable [14, 37]. Consideration of unnecessary radiation is needed when assessing the cost benefit of imaging. We estimated 34 women in our study would receive lumbar spine imaging per year during 2011–2015, but 530 women had lumbar spine imaging each year (radiography 364, CT 139, MRI 79), suggesting significant low value care. We estimated that if clinical guidelines were followed, the cost saving would be over 44 million Australian dollars in Australian women aged 30–39 years over 5 years. This needs to be taken in context of the Australian population where women of this age represent 11.9% of Australian adults aged 20–65 years. The costs of lumbar spine imaging for Australian men and women aged 20–65 years in 2011–2015 would be estimated to be over 434.7 million Australian dollars, which was approximately 0.5% of expenditure on Medicare during 2011–2015 in Australia [38]. This is important information that extends current knowledge, providing policy makers with high quality information on the potential for cost savings to free resources for high value care. Patient expectations, practitioner beliefs, financial incentives, defensive medicine, and time constraint of clinicians may contribute to the discordance between current practice and guidelines and will need to be explored [15].

In our study, depression and poorer physical health were associated with imaging utilization in women with back pain, with no difference in types of imaging modality. Better characterising women at risk of excessive imaging will enable practitioners’ awareness in their care to reduce imaging and offer the potential to develop targeted approaches to reduce low value care. Both patients and clinicians should be targeted for intervention to reduce unnecessary imaging for LBP, as there is evidence of erroneous beliefs from patients [39–41] and clinicians [42, 43] that imaging is necessary in the management of LBP, contributing to the overutilization of imaging investigations.

Our study has limitations. As general practitioners cannot order MBS-funded lumbar spine MRI, all MRIs recorded were requested from non-general practice settings. Imaging procedures, such as MRIs performed privately, inpatient imaging procedures, and MRIs performed on machines that cannot claim a Medicare rebate, may not be recorded in the MBS database, resulting in an underestimation of imaging utilization and associated costs. Women who consented to data linkage (94.5% of the 1973–1978 cohort) had higher education levels and better self-rated health than non-consenters [44]. This might introduce a potential selection bias towards women with higher socioeconomic status and better health, which would have underestimated imaging utilization and associated costs. While our study identified patient characteristics associated with imaging utilization, clinical characteristics, such as the frequency and intensity of back pain and the presence of red flags, could not be assessed due to the unavailability of the data, and data were not available to investigate clinician characteristics of those requesting imaging. This is an area for future research, as clinicians should be targeted for education to reduce low value care. The costs of imaging were calculated over 20 years at 2015 prices, which is likely to overestimate the costs from previous years. Strengths of our study are linking data from a well-established cohort with comprehensive data collected at 3-year intervals over 20 years to the MBS database, and examining imaging use, associated costs and person-level factors over long term.

Lumbar spine imaging was common in population-based Australian women born in 1973–1978 with 38.9% having some form of imaging over 20 years and rates increasing from 1996 to 2015, explained by effect of ageing and time period. Depression and poor physical health were associated with imaging. Targeting women with these conditions and raising awareness of this in clinicians are likely to result in significant cost savings if clinical guidelines are followed, with the potential of freeing resources for high value care and health outcomes.

## Supporting information

S1 Table Numbers of women with different numbers of lumbar spine imaging procedures from 1996 to 2015. (DOCX)

S1 File STROBE statement—checklist of items that should be included in reports of *cohort studies*. (DOCX)

## References

[1] Hoy D, March L, Brooks P, Blyth F, Woolf A, Bain C, et al: The global burden of low back pain: estimates from the Global Burden of Disease 2010 study. Ann Rheum Dis 2014, 73:968–974. 10.1136/annrheumdis-2013-204428 24665116

[2] Balague F, Mannion AF, Pellise F, Cedraschi C: Non-specific low back pain. Lancet 2012, 379:482–491. 10.1016/S0140-6736(11)60610-7 21982256

[3] Downie A, Hancock M, Jenkins H, Buchbinder R, Harris I, Underwood M, et al: How common is imaging for low back pain in primary and emergency care? Systematic review and meta-analysis of over 4 million imaging requests across 21 years. Br J Sports Med 2019 10.1136/bjsports-2018-100087 30760458

[4] Chou R, Qaseem A, Snow V, Casey D, Cross JT Jr., Shekelle P, et al: Diagnosis and treatment of low back pain: a joint clinical practice guideline from the American College of Physicians and the American Pain Society. Ann Intern Med 2007, 147:478–491. 10.7326/0003-4819-147-7-200710020-00006 17909209

[5] National Institute for Health and Care Excellence. 11 2016 Low back pain and sciatica in over 16s: assessment and management NICE guideline NG59 27929617

[6] Toward Optimized Practice (TOP) Low Back Pain Working Group. 2015 12 Evidence-informed primary care management of low back pain: Clinical practice guideline Edmonton, AB: Toward Optimized Practice Available from: http://www.topalbertadoctors.org/cpgs/885801.

[7] NSW Agency for Clinical Innovation. Management of people with acute low back pain: model of care Chatswood; NSW Health; 2016 39 p.

[8] Cassel CK, Guest JA: Choosing wisely: helping physicians and patients make smart decisions about their care. Jama 2012, 307:1801–1802. 10.1001/jama.2012.476 22492759

[9] RANZCR Choosing Wisely Recommendations and Clinical Decision Rules. 2015 http://www.choosingwisely.org.au/getmedia/59b0d1ff-afd8-4abe-8f9e-199431680f74/RANZCR-Clinical-Decision-Rules.pdf.aspx.

[10] Wisely Choosing. American College of Physicians. Five Things Physicians and Patients Should Question.

[11] The "top 5" lists in primary care: meeting the responsibility of professionalism. Arch Intern Med 2011, 171:1385–1390. 10.1001/archinternmed.2011.231 21606090

[12] Chou R, Fu R, Carrino JA, Deyo RA: Imaging strategies for low-back pain: systematic review and meta-analysis. Lancet 2009, 373:463–472. 10.1016/S0140-6736(09)60172-0 19200918

[13] Kendrick D, Fielding K, Bentley E, Kerslake R, Miller P, Pringle M: Radiography of the lumbar spine in primary care patients with low back pain: randomised controlled trial. Bmj 2001, 322:400–405. 10.1136/bmj.322.7283.400 11179160 PMC26570

[14] Chou R, Qaseem A, Owens DK, Shekelle P: Diagnostic imaging for low back pain: advice for high-value health care from the American College of Physicians. Ann Intern Med 2011, 154:181–189. 10.7326/0003-4819-154-3-201102010-00008 21282698

[15] Chou R, Deyo RA, Jarvik JG: Appropriate use of lumbar imaging for evaluation of low back pain. Radiol Clin North Am 2012, 50:569–585. 10.1016/j.rcl.2012.04.005 22643385

[16] Jenkins HJ, Hancock MJ, French SD, Maher CG, Engel RM, Magnussen JS: Effectiveness of interventions designed to reduce the use of imaging for low-back pain: a systematic review. Cmaj 2015, 187:401–408. 10.1503/cmaj.141183 25733741 PMC4387031

[17] Mafi JN, McCarthy EP, Davis RB, Landon BE: Worsening trends in the management and treatment of back pain. JAMA Intern Med 2013, 173:1573–1581. 10.1001/jamainternmed.2013.8992 23896698 PMC4381435

[18] Deyo RA, Mirza SK, Turner JA, Martin BI: Overtreating chronic back pain: time to back off? J Am Board Fam Med 2009, 22:62–68. 10.3122/jabfm.2009.01.080102 19124635 PMC2729142

[19] Fillingim RB, King CD, Ribeiro-Dasilva MC, Rahim-Williams B, Riley JL 3rd: Sex, gender, and pain: a review of recent clinical and experimental findings. J Pain 2009, 10:447–485. 10.1016/j.jpain.2008.12.001 19411059 PMC2677686

[20] Schneider S, Randoll D, Buchner M: Why do women have back pain more than men? A representative prevalence study in the federal republic of Germany. Clin J Pain 2006, 22:738–747. 10.1097/01.ajp.0000210920.03289.93 16988571

[21] Walker BF, Muller R, Grant WD: Low back pain in Australian adults. health provider utilization and care seeking. J Manipulative Physiol Ther 2004, 27:327–335. 10.1016/j.jmpt.2004.04.006 15195040

[22] Lee C, Dobson AJ, Brown WJ, Bryson L, Byles J, Warner-Smith P, et al: Cohort Profile: the Australian Longitudinal Study on Women's Health. Int J Epidemiol 2005, 34:987–991. 10.1093/ije/dyi098 15894591

[23] Brilleman SL, Pachana NA, Dobson AJ: The impact of attrition on the representativeness of cohort studies of older people. BMC Med Res Methodol 2010, 10:71 10.1186/1471-2288-10-71 20687909 PMC2927605

[24] Hockey R, Tooth L, Dobson A: Relative survival: a useful tool to assess generalisability in longitudinal studies of health in older persons. Emerg Themes Epidemiol 2011, 8:3 10.1186/1742-7622-8-3 21294918 PMC3039541

[25] Brown WJ, Bryson L, Byles JE, Dobson AJ, Lee C, Mishra G, et al: Women's Health Australia: recruitment for a national longitudinal cohort study. Women Health 1998, 28:23–40. 10.1300/j013v28n01_03 10022055

[26] Australian Government Department of Health. Madicare Benefits Schedule, MBS Online. http://www.mbsonline.gov.au/internet/mbsonline/publishing.nsf/Content/downloads

[27] Brady SR, Hussain SM, Brown WJ, Heritier S, Billah B, Wang Y, et al: Relationships Between Weight, Physical Activity, and Back Pain in Young Adult Women. Medicine (Baltimore) 2016, 95:e3368 10.1097/MD.0000000000003368 27175634 PMC4902476

[28] Ware JE Jr., Sherbourne CD: The MOS 36-item short-form health survey (SF-36). I. Conceptual framework and item selection. Med Care 1992, 30:473–483. 1593914

[29] Australian Bureau of Statistics, Census of Population and Housing. http://www.abs.gov.au/websitedbs/censushome.nsf/4a256353001af3ed4b2562bb00121564/census.

[30] Williams CM, Maher CG, Hancock MJ, McAuley JH, McLachlan AJ, Britt H, et al: Low back pain and best practice care: A survey of general practice physicians. Arch Intern Med 2010, 170:271–277. 10.1001/archinternmed.2009.507 20142573

[31] Tan A, Zhou J, Kuo YF, Goodwin JS: Variation among Primary Care Physicians in the Use of Imaging for Older Patients with Acute Low Back Pain. J Gen Intern Med 2016, 31:156–163. 10.1007/s11606-015-3475-3 26215847 PMC4720657

[32] Deyo RA, Weinstein JN: Low back pain. N Engl J Med 2001, 344:363–370. 10.1056/NEJM200102013440508 11172169

[33] Bardin LD, King P, Maher CG: Diagnostic triage for low back pain: a practical approach for primary care. Med J Aust 2017, 206:268–273. 10.5694/mja16.00828 28359011

[34] Henschke N, Maher CG, Refshauge KM, Herbert RD, Cumming RG, Bleasel J, et al: Prevalence of and screening for serious spinal pathology in patients presenting to primary care settings with acute low back pain. Arthritis Rheum 2009, 60:3072–3080. 10.1002/art.24853 19790051

[35] Enthoven WT, Geuze J, Scheele J, Bierma-Zeinstra SM, Bueving HJ, Bohnen AM, et al: Prevalence and "Red Flags" Regarding Specified Causes of Back Pain in Older Adults Presenting in General Practice. Phys Ther 2016, 96:305–312. 10.2522/ptj.20140525 26183589

[36] Jarvik JG, Deyo RA: Diagnostic evaluation of low back pain with emphasis on imaging. Ann Intern Med 2002, 137:586–597. 10.7326/0003-4819-137-7-200210010-00010 12353946

[37] Davis PC, Wippold FJ 2nd, Brunberg JA, Cornelius RS, De La Paz RL, Dormont PD, et al: ACR Appropriateness Criteria on low back pain. J Am Coll Radiol 2009, 6:401–407. 10.1016/j.jacr.2009.02.008 19467485

[38] Finance Budget 2013–14 Budget papers No. 1—Budget strategy and outlook Commonwealth of Australia 2013.

[39] Hoffmann TC, Del Mar CB, Strong J, Mai J: Patients' expectations of acute low back pain management: implications for evidence uptake. BMC Fam Pract 2013, 14:7 10.1186/1471-2296-14-7 23297840 PMC3544646

[40] Jenkins HJ, Hancock MJ, Maher CG, French SD, Magnussen JS: Understanding patient beliefs regarding the use of imaging in the management of low back pain. Eur J Pain 2016, 20:573–580. 10.1002/ejp.764 26282178

[41] Chou L, Ranger TA, Peiris W, Cicuttini FM, Urquhart DM, Sullivan K, et al: Patients' perceived needs for medical services for non-specific low back pain: A systematic scoping review. PLoS One 2018, 13:e0204885 10.1371/journal.pone.0204885 30408039 PMC6224057

[42] Buchbinder R, Staples M, Jolley D: Doctors with a special interest in back pain have poorer knowledge about how to treat back pain. Spine (Phila Pa 1976) 2009, 34:1218–1226; discussion 1227. 19407674 10.1097/BRS.0b013e318195d688

[43] Slade SC, Kent P, Patel S, Bucknall T, Buchbinder R: Barriers to Primary Care Clinician Adherence to Clinical Guidelines for the Management of Low Back Pain: A Systematic Review and Metasynthesis of Qualitative Studies. Clin J Pain 2016, 32:800–816. 10.1097/AJP.0000000000000324 26710217

[44] Australian Longitudinal Study of Women’s Health: Technical Report No. 29. In Australian Government Department of Health and Ageing Brisbane: Australian Longitudinal Study of Women’s Health; 12 2007 Available from: https://www.alswh.org.au/images/content/pdf/technical_reports/report_29_alswh.pdf

