# Supplementary material for: Rates, costs and determinants of lumbar spine imaging in population-based women born in 1973–1978: Data from the Australian Longitudinal Study on Women’s Health
Source: PLoS One. 2020 Dec 3;15(12):e0243282. doi: 10.1371/journal.pone.0243282 (PMC7714143; doi:10.1371/journal.pone.0243282)
Supplement: S1 Table — (DOCX) [file pone.0243282.s001.docx]

**S1 Table. Numbers of women with different numbers of lumbar spine imaging procedures from 1996 to 2015**

| **No of imaging procedures** | **Any imaging**  **N=5237** | **Radiography**  **N=4648** | **CT**  **N=1413** | **MRI**  **N=556** |
| --- | --- | --- | --- | --- |
| 1 | 2991 | 3097 | 1093 | 424 |
| 2 | 1193 | 1022 | 220 | 82 |
| 3 | 544 | 349 | 68 | 20 |
| 4 | 244 | 106 | 19 | 15 |
| 5 | 110 | 38 | 7 | 3 |
| 6 | 65 | 15 | 2 | 7 |
| 7 | 22 | 9 | 1 | 2 |
| 8 | 21 | 4 | 1 | 1 |
| 9 | 21 | 3 |  | 2 |
| 10 | 6 | 2 | 2 |  |
| 11 | 7 | 2 |  |  |
| 12 | 3 |  |  |  |
| 13 | 4 |  |  |  |
| 15 | 2 |  |  |  |
| 16 | 2 | 1 |  |  |
| 22 | 1 |  |  |  |
| 27 | 1 |  |  |  |

CT: computed tomography; MRI: magnetic resonance imaging
